# A Rare Case of Intravascular Large B-cell Lymphoma Presenting as Leukemic Phase in a Patient With HCV-Related Cirrhosis

**DOI:** 10.7759/cureus.87705

**Published:** 2025-07-11

**Authors:** Michael Assefa

**Affiliations:** 1 Internal Medicine, Northside Hospital Cherokee, Canton, USA

**Keywords:** aggressive b cell lymphoma, cirrhosis-associated immune dysfunction, decompensated cirrhosis, diffuse large b cell lymphoma, hcv-related cirrhosis, intravascular lymphoma, leukemic phase presentation, non-hodgkin lymphoma, peripheral smear, rare lymphoma

## Abstract

We report a rare case of a 65-year-old man with decompensated cirrhosis due to alcohol and HCV, who presented with altered mental status, hypotension, and a diffuse rash. Laboratory findings were notable for leukocytosis, severe coagulopathy, and acute kidney injury. Peripheral smear and flow cytometry confirmed intravascular large B-cell lymphoma (IVLBCL), a rare and aggressive subtype of non-Hodgkin lymphoma characterized by intravascular proliferation of malignant lymphocytes. Even more rarely, the patient presented with a leukemic phase, marked by circulating lymphoma cells, a presentation reported in only a few cases worldwide. The diagnostic challenge was further compounded by liver dysfunction, as features of advanced cirrhosis, such as coagulopathy, encephalopathy, and renal injury closely overlapped with lymphoma-related findings. This case underscores the need for a broad differential diagnosis in cirrhotic patients with multiorgan dysfunction and highlights how systemic illness can obscure and delay the recognition of aggressive malignancies.

## Introduction

Intravascular large B-cell lymphoma (IVLBCL) is a rare, aggressive subtype of diffuse large B-cell lymphoma, characterized by the proliferation of malignant lymphocytes within the lumina of small vessels, particularly capillaries and post-capillary venules, without forming a discrete tumor mass or causing lymphadenopathy [1]. It often presents with nonspecific systemic symptoms and is frequently diagnosed post-mortem. In Western populations, IVLBCL commonly involves the skin and central nervous system [2]. According to population-based data from the Surveillance, Epidemiology, and End Results (SEER) Program and the National Cancer Database from 2000 to 2013, the incidence of IVLBCL is approximately 0.095 cases per 1,000,000 persons per year in the United States [3]. The leukemic-phase presentation of IVLBCL corresponds to Stage IV disease and is characterized by circulating neoplastic lymphoid cells in the peripheral blood. This presentation is exceedingly rare and has been described only in isolated case reports and small series [4]. We present a diagnostically challenging case of IVLBCL presenting in the leukemic phase in a patient with HCV-related decompensated cirrhosis. The underlying malignancy was obscured by overlapping features of advanced liver disease, including coagulopathy, encephalopathy, and renal dysfunction, making the timely recognition of the lymphoma more difficult.

## Case presentation

A 65-year-old man with a history of decompensated cirrhosis due to alcohol use and chronic HCV infection, hypertension, type 2 diabetes mellitus, obstructive sleep apnea, and seizure disorder presented to the emergency department with confusion and hypotension. He had been recently evaluated in the hepatology clinic and was on the wait list for liver transplantation. His wife reported the onset of a petechial facial rash over the previous four to five days, accompanied by increasing lethargy. He had also recently returned from a vacation on the Mississippi River.

**Figure 1:**
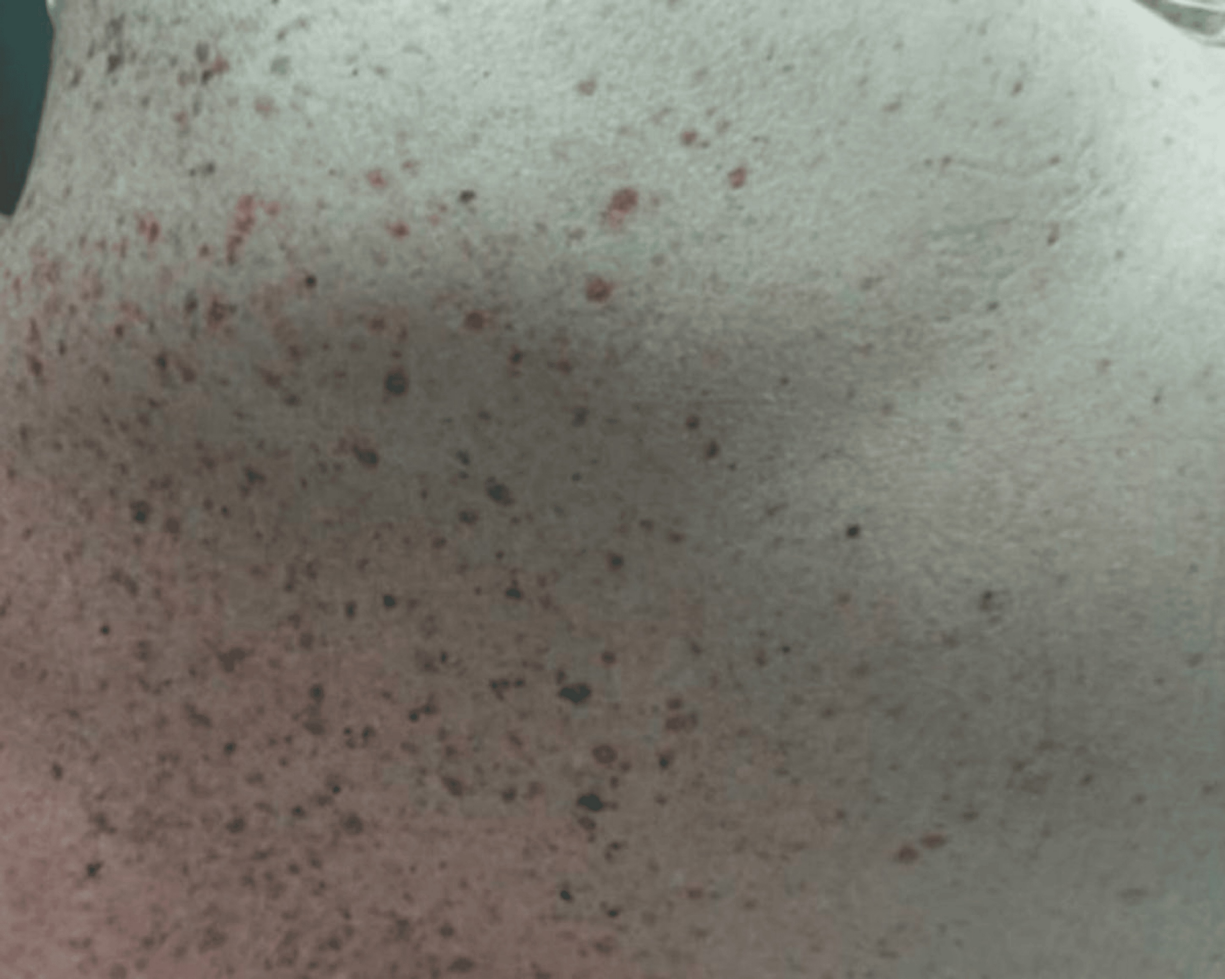
Diffuse papular rash on torso A non-blanching, erythematous, coalescing papular rash observed over the back on initial physical examination.

On examination, the patient was hypotensive (blood pressure 94/47 mmHg), afebrile, and had a heart rate of 70 and a respiratory rate of 20. A diffuse, non-blanching, non-pruritic, erythematous maculopapular rash, consisting of flat erythematous areas interspersed with raised, coalescing papules, was noted across his face, chest, and back (Figure 1). Neurologically, he was intermittently confused and responded slowly.

Initial laboratory evaluation demonstrated profound cytopenias, coagulopathy, renal and hepatic dysfunction, and severe metabolic disturbances (Table 1). Laboratory tests revealed marked leukocytosis (white blood cell count 47×10⁹/L), anemia (hemoglobin 10.2 g/dL), and thrombocytopenia (platelet count 42,000/μL). Coagulation studies were consistent with disseminated intravascular coagulation, showing an international normalized ratio of 3.6, a partial thromboplastin time of 44 seconds, D-dimer >20 μg/mL, positive fibrin monomer, and a decline in fibrinogen levels from 140 to 88 mg/dL. Metabolic panels indicated acute kidney injury (creatinine 3.63 mg/dL, blood urea nitrogen 80 mg/dL), progressive lactic acidosis (lactate rising from 11.4 to 18 mmol/L), elevated uric acid (10.1 mg/dL), and hyperammonemia (62 μmol/L). Liver function tests showed rising bilirubin (from 9.2 to 9.9 mg/dL), aspartate aminotransferase decreasing from 447 to 379 U/L, alanine aminotransferase of 73 U/L, and alkaline phosphatase declining from 171 to 137 U/L. The calculated model for the end-stage liver disease score was 38.

**Table 1:** Laboratory findings on admission

Laboratory Test	Patient Value	Reference Range
White blood cell count	47×10⁹/L	4.0-10.0×10⁹/L
Hemoglobin level	10.2 g/dL	13.5-17.5 g/dL (male)
Platelet count	42×10⁹/L	150-450×10⁹/L
International normalized ratio	3.6	0.9-1.2
Partial thromboplastin time	44 seconds	25-35 seconds
D-dimer level	>20 μg/mL	<0.5 μg/mL
Fibrin monomer test	Positive	Negative
Fibrinogen level	140 mg/dL	200-400 mg/dL
Serum creatinine	3.63 mg/dL	0.6-1.3 mg/dL
Blood urea nitrogen	80 mg/dL	7-20 mg/dL
Serum lactate	11.4 mmol/L	0.5-2.2 mmol/L
Serum uric acid	10.1 mg/dL	3.5-7.2 mg/dL
Serum ammonia	62 μmol/L	15-45 μmol/L
Total bilirubin	9.2 mg/dL	0.1-1.2 mg/dL
Aspartate aminotransferase	447 U/L	10-40 U/L
Alanine aminotransferase	73 U/L	7-56 U/L
Alkaline phosphatase	171 U/L	44-147 U/L

The patient was admitted to the intensive care unit with a working diagnosis of septic or distributive shock, acute kidney injury, disseminated intravascular coagulation, and hepatic encephalopathy. Management included broad-spectrum antibiotics, intravenous fluids, albumin, bicarbonate infusion for metabolic acidosis, dextrose for hypoglycemia, and cryoprecipitate for hypofibrinogenemia. Rifaximin was added to his home lactulose regimen. Paracentesis for ascites revealed a transudative profile. Continuous renal replacement therapy was initiated. Infectious workup, including blood, urine, and ascitic fluid cultures, was negative. There was no clinical or laboratory evidence of an acute hepatitis C flare at the time of presentation.

**Figure 2:**
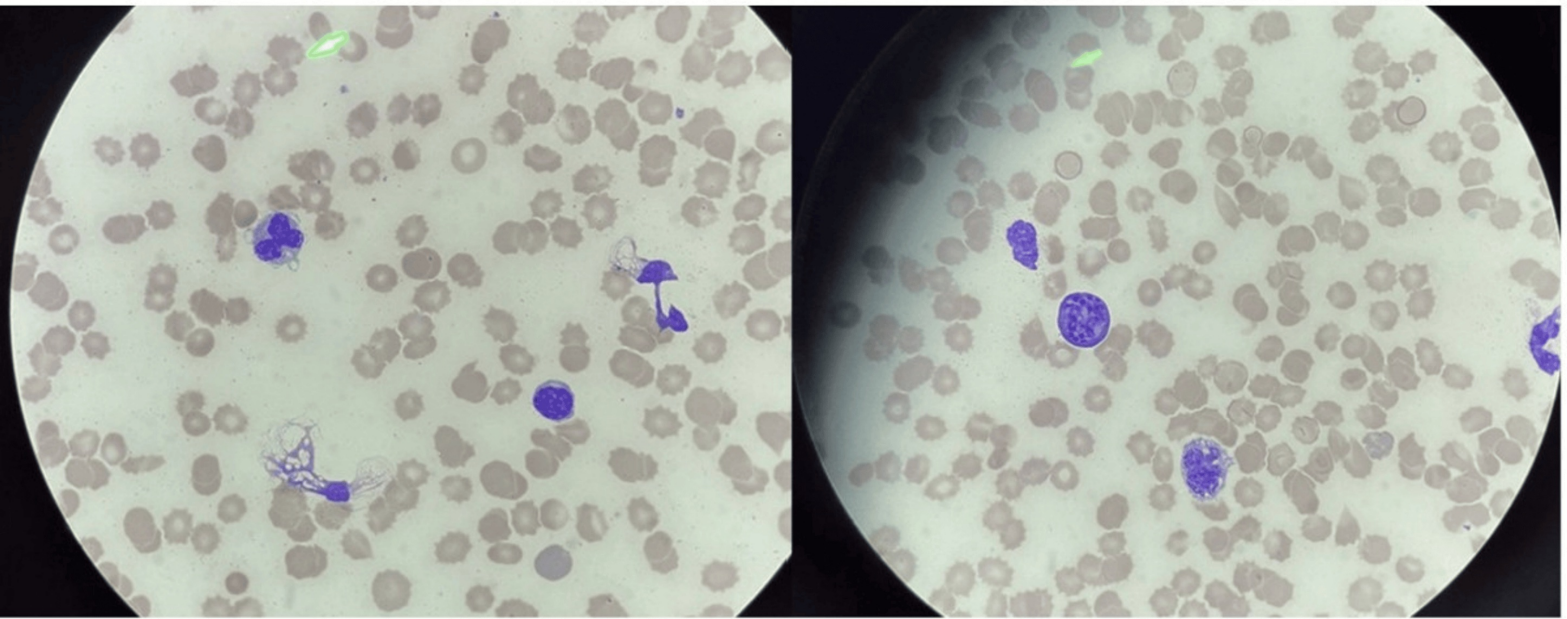
Peripheral blood smear showing abnormal lymphocytes Peripheral smear demonstrates circulating atypical lymphocytes with irregular nuclear contours and scant cytoplasm, consistent with intravascular large B-cell lymphoma (IVLBCL).

Peripheral smear revealed abnormal circulating lymphocytes, and flow cytometry confirmed aggressive B-cell lymphoma consistent with IVLBCL (Figure 2).

Although his hemodynamics briefly improved with resuscitation, his neurologic function declined, and he became obtunded. The diagnosis rendered him ineligible for liver transplantation. Plans for rasburicase and dexamethasone were discussed, but the family ultimately elected comfort care. The patient passed away a few days after presentation.

## Discussion

IVLBCL is a pathologically distinct, clinically aggressive, and exceedingly rare subtype of extranodal large B-cell lymphoma defined by the selective growth of malignant lymphocytes within the lumina of small blood vessels. It typically presents with systemic symptoms such as B symptoms, cytopenias, and neurologic or cutaneous involvement, often without detectable lymphadenopathy, factors that contribute to diagnostic delay and poor outcomes in many cases [1,2].

Although the true incidence of IVLBCL remains unknown [4], a population-based study using U.S. SEER and National Cancer Database (NCDB) data estimated an age-adjusted incidence of 0.095 cases per 1,000,000 from 2000 to 2013, with a significant annual increase of 9.84% [5]. Despite its intravascular localization, a leukemic phase, characterized by abundant circulating lymphoma cells, is extremely rare. A retrospective review from MD Anderson Cancer Center identified only six such cases, each with ≥10% lymphoma cells in peripheral blood, often with bone marrow involvement, CD5 positivity, complex cytogenetics, and high mortality [6]. Recognition of this rare presentation expands the clinicopathologic spectrum of IVLBCL and underscores its diagnostic challenge.

Cirrhosis, particularly due to chronic HCV infection, further complicates the clinical picture. The overlap of cirrhosis-related features, such as encephalopathy, cytopenias, coagulopathy, and multiorgan dysfunction, may delay the recognition of an underlying hematologic malignancy. Cirrhosis is associated with chronic immune dysregulation, impaired clearance of malignant clones, and systemic inflammation, all of which can increase the risk of lymphoproliferative disorders [7,8]. This immune dysfunction, often referred to as cirrhosis-associated immune dysfunction syndrome (CAIDS), involves both immune suppression and persistent inflammation that impair immune surveillance [9].

In patients with HCV-related cirrhosis, the risk of B-cell non-Hodgkin lymphoma is further elevated due to direct lymphotropic effects of the virus [10]. In this case, the patient’s clinical decline was initially attributed to hepatic decompensation and presumed sepsis. However, the degree of leukocytosis, elevated lactate and uric acid levels, and atypical cutaneous findings prompted hematologic investigation. The presence of numerous circulating lymphoma cells likely reflected a leukemic variant of IVLBCL, an exceedingly rare manifestation.

This case reinforces the importance of maintaining a broad differential diagnosis in patients with cirrhosis and systemic decline. It also highlights how cirrhosis can obscure signs of aggressive malignancies, contributing to diagnostic delay and limiting treatment options.

## Conclusions

IVLBCL is a rare and aggressive malignancy that may present atypically with circulating disease mimicking a leukemic phase. This unusual presentation, coupled with overlapping features of hepatic decompensation and systemic inflammation, makes timely diagnosis especially challenging in patients with cirrhosis.

This case emphasizes the importance of maintaining a broad differential diagnosis when evaluating cirrhotic patients with multiorgan dysfunction, cytopenias, or unexplained systemic decline. Early consideration of hematologic malignancy and prompt hematopathologic investigation can be critical to avoid delays in diagnosis and missed opportunities for treatment.
